# Radial entry for ESIN fixation of pediatric radial fractures: a 10-year cohort study with focus on neurological complications

**DOI:** 10.1007/s00590-026-04782-2

**Published:** 2026-05-22

**Authors:** Maximilian Leiblein, Lilith Van Groeninghen, Lewin-Caspar Busse, Ingo Marzi, Katharina Sommer

**Affiliations:** Department of Trauma Surgery and Orthopedics, University Hospital, Goethe University, Frankfurt, Germany

**Keywords:** ESIN, Pediatric Radial Fracture, Radial Nerve, Complication

## Abstract

**Background:**

Elastic stable intramedullary nailing (ESIN) is an established treatment for pediatric radius fractures. However, the optimal surgical entry point remains debated due to differing complication profiles. The radial approach carries a risk of superficial branch of the radial nerve (SBRN) irritation, whereas dorsal approaches have been associated with extensor tendon injuries. This study evaluates the neurological complication profile of the radial approach and provides clinically relevant risk factors.

**Methods:**

A retrospective single-center study was performed including 285 pediatric patients treated with ESIN for radius fractures between 2015 and 2024 using a standardized radial entry technique. Neurological complications were assessed at predefined time points. Associations between patient age, fracture characteristics, entry point location, and neurological outcomes were analyzed.

**Results:**

New SBRN lesions occurred in 12 of 285 patients (4.2%) after ESIN implantation and in 3 patients (1.1%) after implant removal. Most deficits were transient, while persistent deficits were observed in 2.1% of cases. A more proximal entry point showed a trend toward increased risk of SBRN injury (*p* = 0.055), with a potential threshold at approximately 18 mm from the distal radial physis. Older patient age was significantly associated with increased risk of neurological complications (*p* < 0.001). No extensor pollicis longus (EPL) tendon ruptures were observed.

**Conclusion:**

Radial entry ESIN is associated with a low rate of predominantly transient neurological complications. Entry point positioning and patient age appear to influence the risk of SBRN injury. Based on our data and the available literature, the radial approach represents a safe and clinically favorable option for ESIN of pediatric radius fractures.

## Introduction

 Metaphyseal and proximal radius fractures are among the most common injuries in children [1–5]. For unstable or displaced fractures, elastic stable intramedullary nailing (ESIN) has become an established minimally invasive and effective treatment modality [6, 7]. Despite its widespread use, there is ongoing debate regarding the optimal surgical entry point for nail insertion [8–10]. Both the radial and dorsal approaches are commonly performed, yet they differ significantly in their respective risk profiles [11].

The radial approach provides a direct orientation to the fracture line and facilitates precise implant positioning. However, the superficial branch of the radial nerve (SBRN) lies in close proximity to the entry site, placing it at risk of irritation or injury. Damage to the SBRN may result in sensory disturbances, dysesthesia, and pain over the dorsoradial aspect of the hand, potentially impairing hand function.

In contrast, the dorsal approach avoids the risk of SBRN injury but introduces a different set of hazards. Of particular concern is the potential iatrogenic injury to the extensor pollicis longus (EPL) tendon [8]. EPL rupture results in loss of active thumb extension at the interphalangeal joint and typically necessitates surgical reconstruction.

Given these competing risks, high-quality data comparing the complication profiles of the two entry points are needed. The present retrospective study evaluates complications associated with the radial approach in ESIN treatment of meta-diaphyseal, diaphyseal and proximal radius fractures in children, based on a 10-year cohort from a Level I trauma center in Germany. Particular focus is placed on the occurrence, severity, and clinical relevance of SBRN irritation. The findings are compared with published data on complications associated with the dorsal approach, especially the incidence of EPL injuries.

The objective of this study is to evaluate the neurological complication profile of the radial approach in ESIN of pediatric radius fractures and to contextualize these findings in relation to published data on dorsal entry techniques.

## Materials and methods

### Study design and ethical approval

This retrospective single-center study was approved by the local institutional ethics committee. All procedures were performed in accordance with the ethical standards of the institutional and national research committee and with the Declaration of Helsinki.

All pediatric patients who underwent elastic stable intramedullary nailing (ESIN) of the radius between January 2015 and December 2024 were identified and retrospectively reviewed.

### Inclusion criteria

Patients younger than 18 years with meta-diaphyseal, diaphyseal, or proximal radius fractures treated operatively using ESIN via a radial entry point were eligible for inclusion. ESIN was also applied in selected adolescent patients up to the age of 17 years depending on fracture characteristics. Fracture diagnosis and classification were confirmed using standard anteroposterior (AP) and lateral radiographs.

### Exclusion criteria

Exclusion criteria at the patient level included incomplete clinical or radiological documentation, insufficient follow-up, and missing radiographs preventing accurate assessment of the entry point.

Pre-existing neurological deficits were not considered an exclusion criterion at the patient level but were excluded from the analysis of access-related neurological complications in order to avoid confounding by trauma-related nerve injury.

## Data collection

### Demographic variables

The following demographic and injury-related data were extracted: age, sex, and affected side, date and mechanism of trauma.

### Radiographic assessment

Preoperative radiographs were analyzed for fracture location (proximal, middle, or distal third), involvement of the ulna, angulation (in degrees in AP and lateral view), displacement (in millimeters) for complete fractures (in AP and lateral view) and intramedullary canal diameter (in millimeters), measured on standardized AP radiographs.

The distance of the radial ESIN entry point from the distal radial epiphyseal plate was measured in millimeters on standardized AP radiographs. Measurements were performed using calibrated digital imaging software.

Nail diameter and the ratio between nail size and canal width were also calculated.

## Surgical technique

**Fig. 1:**
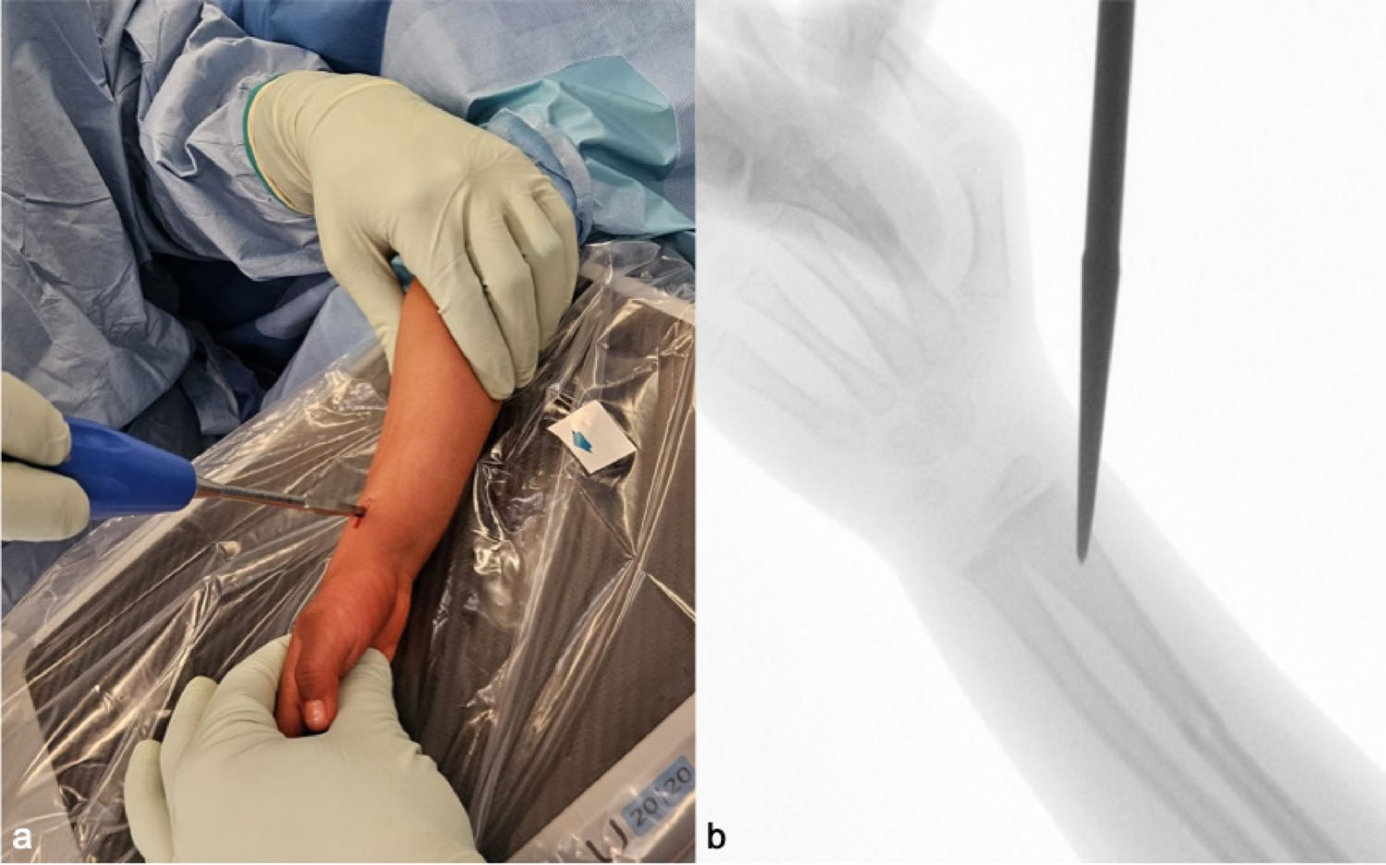
Clinical (**a**) and radiological (**b**) illustration of the radial entry point in relation to the distal radial growth plate. The SBRN was identified and protected prior to awl insertion and is running dorsally to the entry point

All patients underwent ESIN implantation using a standardized radial entry point, located about 1.0–1.5 cm proximal to the distal radial growth plate. The incision (about 10 mm) was positioned on the radial aspect of the radius between the brachioradialis and the extensor carpi radialis longus/brevis tendons (Fig. 1). Care was taken to avoid affection of the Ranvier groove. The superficial branch of the radial nerve (SBRN) typically runs dorsal to the entry site.

Hardware removal was routinely performed approximately six months after implantation using the same surgical approach. A skin incision of 1–2 cm was used to ensure adequate visualization and to minimize the risk of iatrogenic SBRN injury.

## Follow-up and outcome assessment

Patients were followed until implant removal. All complications were recorded, with particular focus on neurological deficits involving the SBRN.

Functional outcomes included active range of motion (ROM) of the wrist, forearm and elbow, and sensory assessment in the SBRN distribution.

Radiographic consolidation was evaluated on follow-up radiographs and documented as an outcome parameter.

### Neurological assessment and outcome categories

Neurological status was documented at three predefined time points: preoperatively, postoperatively after ESIN implantation, and after implant removal. Neurological deficits were categorized according to motoric or sensory deficits and to the affected nerve: Radial nerve with focus on the superficial branch of the radial nerve (SBRN), median nerve, ulnar nerve. Combined deficits (e.g., SBRN / ulnar) were assigned to each involved nerve. For the primary analysis, preoperative neurological deficits were considered access-independent and excluded from access-related risk calculations.

Neurological deficits were further classified as transient if they resolved by the final follow-up after implant removal, or persistent if still present at that time point. The primary outcome was the incidence of new SBRN lesions after ESIN implantation, implant removal, and overall access-related morbidity (ESIN and/or removal). Median and ulnar nerve lesions were analyzed descriptively and reported separately.

### Statistical analysis

Associations between fracture characteristics, entry point location, patient age, and neurological complications were assessed. Continuous variables (entry point distance, angulation, displacement, age) were analyzed using the Mann–Whitney U test due to non-normal distribution. Categorical variables (fracture location, ulnar involvement, neurological outcomes, and open vs. closed fractures) were compared using Chi-square or Fisher’s exact tests as appropriate.

A significance level of *p* < 0.05 was considered statistically significant for all two-sided tests. Statistical analyses and figure generation were performed using standard statistical software.

### Literature research

To compare the complications identified in our cohort with those reported for the dorsal entry point, a focused literature search was conducted. Studies describing complications associated with dorsal-entry ESIN in pediatric radius or forearm fractures were screened and evaluated. Particular attention was given to publications reporting neurological or tendinous complications, especially injuries to the extensor pollicis longus (EPL) tendon. The extracted data served to contextualize the radial-approach–related complications observed in the present study.

## Results

### Demographic data

**Fig. 2:**
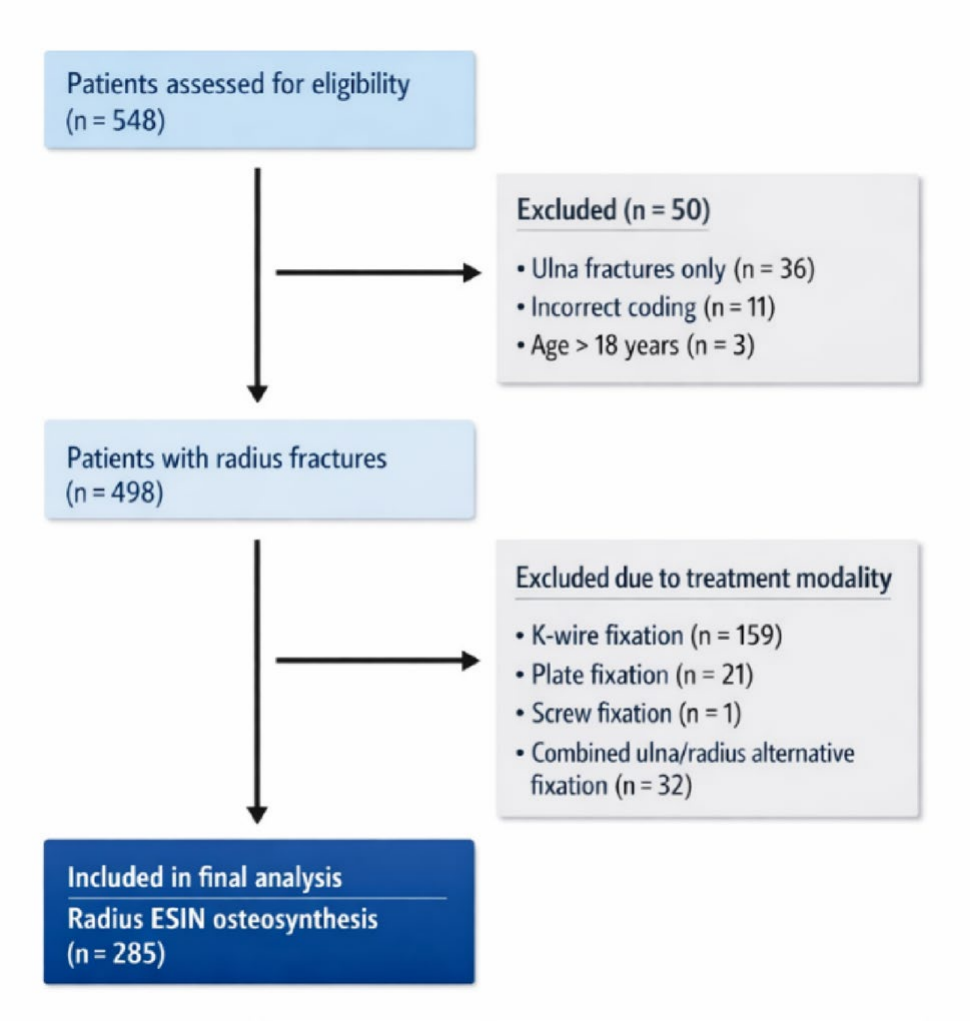
Flowchart of patient selection, illustrating the screening process and study inclusion

In total, we identified 285 patients between January 2015 and December 2024, who met the inclusion criteria (Fig. 2).

Patients showed a mean age of 7.4 years (median 7.0 years), ranging from one to seventeen years. Of the 285 included patients, 183 were male (64.2%) and 102 (35.8%) were female. The right side was affected in 116 (40.7%) cases, the left arm in 169 (59.3%).

### Trauma mechanism

Analyzing the mechanism of trauma, patients were subsumed in four categories: Fall from a standing position or walking (53.7%), fall from height (26.3%), sports accidents (17.9%) and traffic accidents or violence (2.1%).

### Fracture details and treatment

Of the 285 patients, 230 (80.7%) had a fracture in the midshaft, 30 (10.5%) dia-metaphyseal and 25 (8.8%) of the proximal radius. Ulna was involved in 243 cases (85.3%). Evaluation of the angulation of the fractured radius showed a median of 24.2°, ranging from 4.7° to 99.8°. In complete fractures median dislocation was 3.48 mm, ranging from 0.2 to 16.22 mm.

In 276 (96.9%) fractures reduction was done closed, in 9 (3.1%) cases, an open reduction was necessary (Table 1).

**Table 1:** Fracture details and therapy

Fracture location (*n*, %)	Mid: 230 (80.7%) Met.-dia.: 30 (10.5%) Prox: 25 (8.8%)
Ulnar involvement (n, %)	y: 243 (85.3%) n: 42 (14.7%)
Angulation (°)	Median 24.2, Range 4.7–99.8
Dislocation (mm, complete fractures only)	Median 3.48, Range 0.20–16.22, *n* = 96
Open / closed reduction (n, %)	closed: 276 (96.9%) open: 9 (3.1%)
Ratio intramedullary canal / ESIN diameter	Median 1.69, Range 2.91–1.04
Entry point (physis to centre ESIN, mm)	Median 13.74, Range 2.66–35.57
Time to Implant Removal (months)	Median 5, Range 1–11

**Fig. 3:**
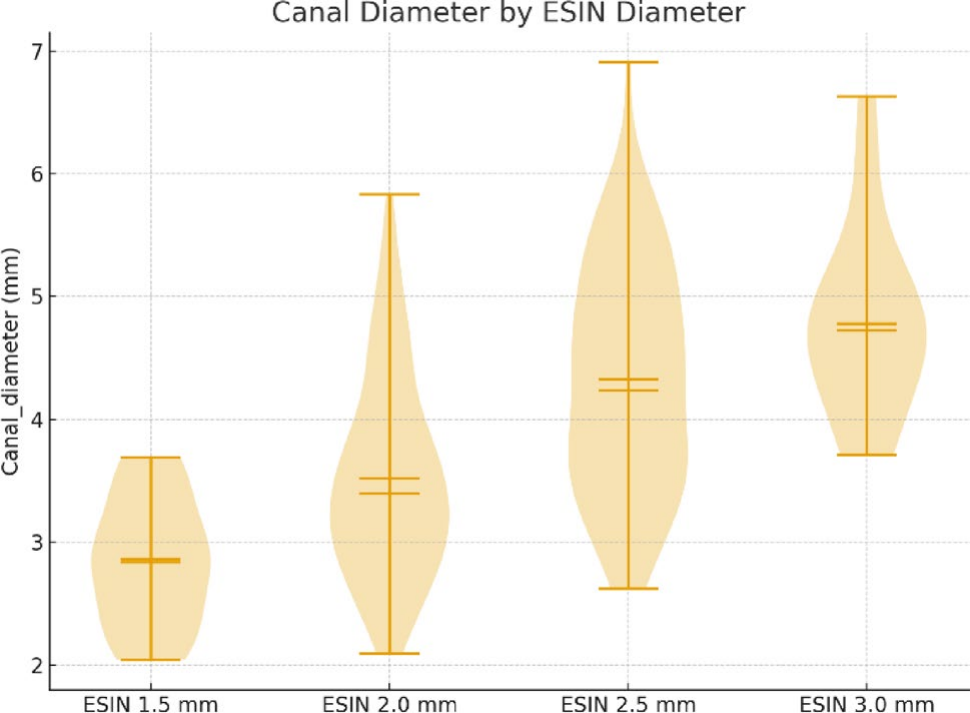
Distribution of intramedullary canal diameter in relation to the selected ESIN diameter. Shown are violin plots with median and mean indicators. Larger canal diameters were associated with the use of thicker ESIN implants, reflecting standard sizing principles in pediatric elastic stable intramedullary nailing

Intramedullary canal diameter was measured in standard AP radiographs and contexted with the respective ESIN-diameter (Fig. 3).

### Neurological complications

Preoperative neurological deficits were documented in a small subset of patients and affected predominantly the ulnar nerve, followed by the superficial radial nerve (SBRN). These baseline findings were excluded from subsequent analyses of access-related risk.

**Fig. 4:**
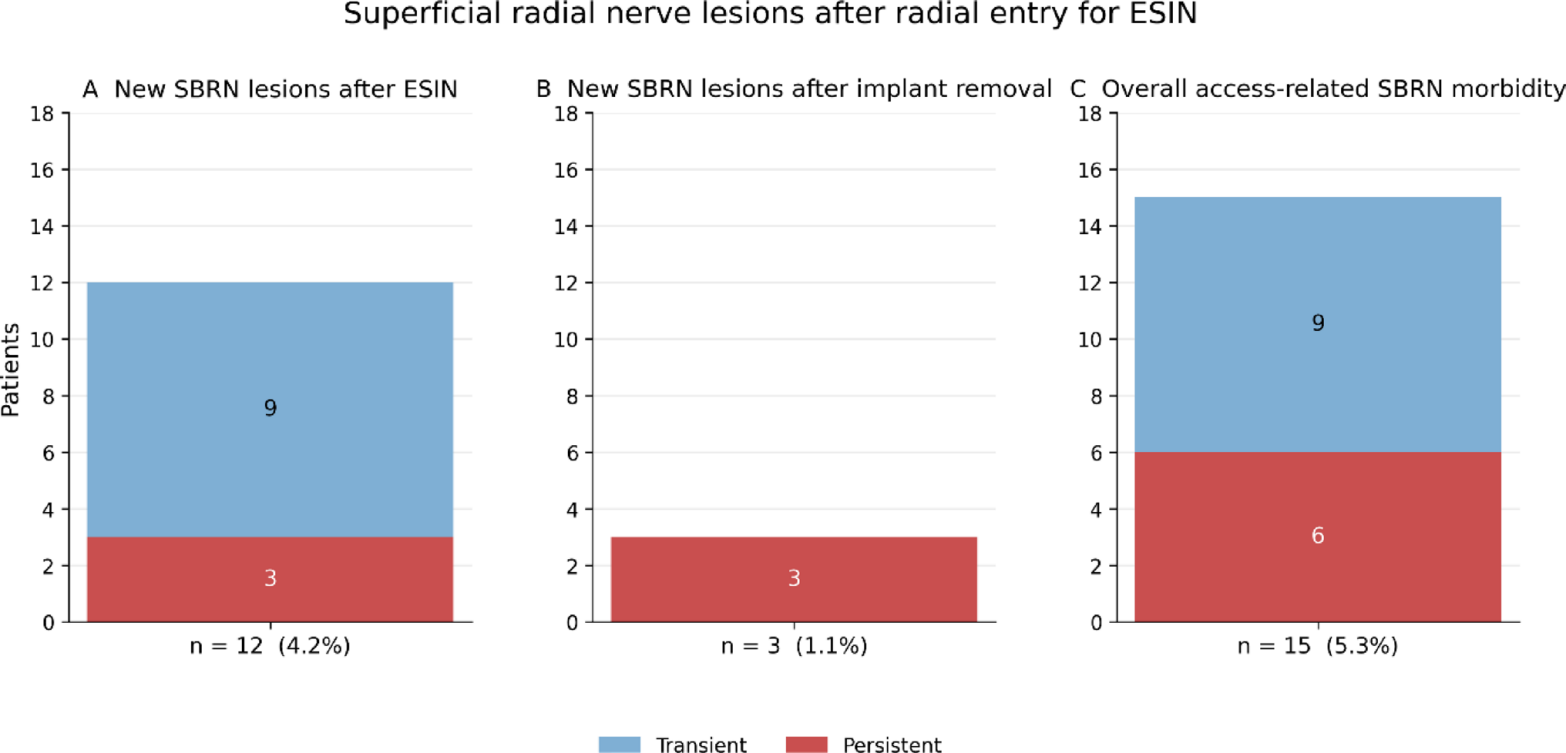
Superficial radial nerve lesions after radial entry. **A** Shows new superficial radial nerve (SBRN) lesions occurring after ESIN implantation. **B** Depicts new SBRN lesions after implant removal. **C** Summarizes the overall access-related SBRN morbidity combining both surgical steps. Transient lesions resolved by final follow-up, whereas persistent lesions were still present at the end of the observation period

After exclusion of baseline deficits, new superficial radial nerve lesions occurred in 12 of 285 patients (4.2%) following ESIN implantation. Of these, 9 deficits were transient and resolved completely by final follow-up, whereas 3 deficits (1.1%) persisted (Fig. 4).

New SBRN lesions following implant removal were observed in 3 patients (1.1%), all of which were persistent at final follow-up. No transient SBRN deficits were recorded after implant removal.

Considering both surgical steps, the overall access-related morbidity of the superficial radial nerve was 15 of 285 patients (5.3%). Of these, 9 deficits were transient and 6 deficits (2.1%) were persistent at final follow-up.

Median and ulnar nerve lesions were rare and occurred almost exclusively as preoperative findings or transient postoperative phenomena (one median, four ulnar after ESIN implantation). No persistent median or ulnar nerve deficits were observed at final follow-up. Preoperatively, neurological deficits were documented in 12 patients (4.2%), including 6 SBRN, 8 ulnar (two patients with symptoms of radial and ulnar nerve), and no median nerve lesions.

Importantly, no extensor pollicis longus (EPL) tendon ruptures were observed in the entire cohort.

### Entry point

Analysis of the radial entry point, measured from the epiphyseal plate to the center of the ESIN in standard AP radiographs showed a median distance of 13.74 mm (range 2.66–35.57 mm).

For the purpose of entry point analysis as factor for SBRN affection, only new-onset SBRN lesions were considered. Patients with any preoperative neurological deficit were excluded from this analysis to avoid confounding by injury-related nerve damage.

New postoperative SBRN lesions were defined as the presence of radial nerve symptoms documented after ESIN implantation in patients with normal preoperative neurological status.

The distance of the entry point from the epiphyseal plate was compared between patients with and without new postoperative SBRN lesions.

**Fig. 5:**
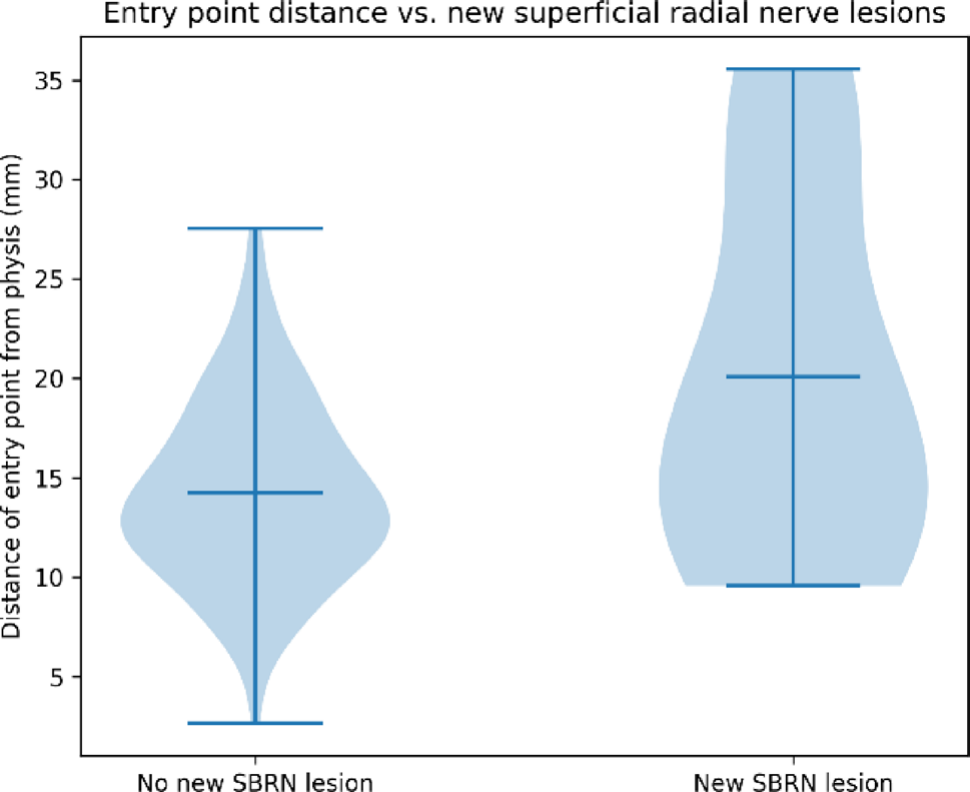
Violin plot illustrating the distance of the ESIN entry point from the distal radial epiphyseal plate (in millimeters) in patients with and without newly developed postoperative superficial radial nerve (SBRN) lesions. Horizontal lines indicate median values, and the width of the violins represents data distribution

Patients who developed a new postoperative SBRN lesion demonstrated a greater median distance between the ESIN entry point and the distal radial epiphyseal plate compared with patients without neurological complications (Fig. 5). This difference showed a clear trend toward statistical significance in the nonparametric comparison (Mann–Whitney U test: *p* = 0.055).

ROC analysis identified an optimal cut-off of approximately 18 mm between the entry point and the distal radial epiphyseal plate for predicting new superficial radial nerve lesions (sensitivity 54.5%, specificity 80.8%). Patients with an entry point located more than 18 mm from the epiphyseal plate showed a markedly higher rate of new SBRN lesions (9.3% vs. 2.8%).

### Ratio IM / ESIN

To assess, whether the size of the nail affects the occurrence of radial nerve lesion, the ratio between intramedullary canal and ESIN-diameter was calculated and put in context with post-operative deficit of the SBRN. No significant correlation could be identified (*p* > 0.05).

### Association between fracture morphology and neurological deficits

The association between fracture morphology and neurological impairment was evaluated across pre-operative, post-ESIN, and post implant removal timepoints. A significant relationship was observed between fracture location and the presence of a primary pre-operative neurological deficit (Chi² test, *p* ≈ 0.005). All primary neurological findings occurred exclusively in patients with midshaft fractures, whereas no proximal or distal fractures in this cohort demonstrated pre-operative nerve involvement. Other morphology-related parameters—including fracture angulation, dislocation in complete fractures — did not show a statistically significant association with pre-operative neurological abnormalities. Also, ulnar involvement was not associated with the occurrence of postoperative neurological deficits. No significant differences were observed between patients with isolated radius fractures and those with combined radius and ulna fractures.

Importantly, none of the assessed fracture characteristics were associated with new neurological deficits occurring after ESIN implantation or after subsequent metal removal. Mann–Whitney U tests for angulation and dislocation, as well as Fisher’s exact tests for categorical variables, consistently yielded non-significant results for postoperative deficits. These findings indicate that while midshaft fractures may carry a higher risk of primary neurological impairment at presentation, fracture morphology does not appear to contribute to procedure-related neurological complications following ESIN treatment or hardware removal.

### Age

**Fig. 6:**
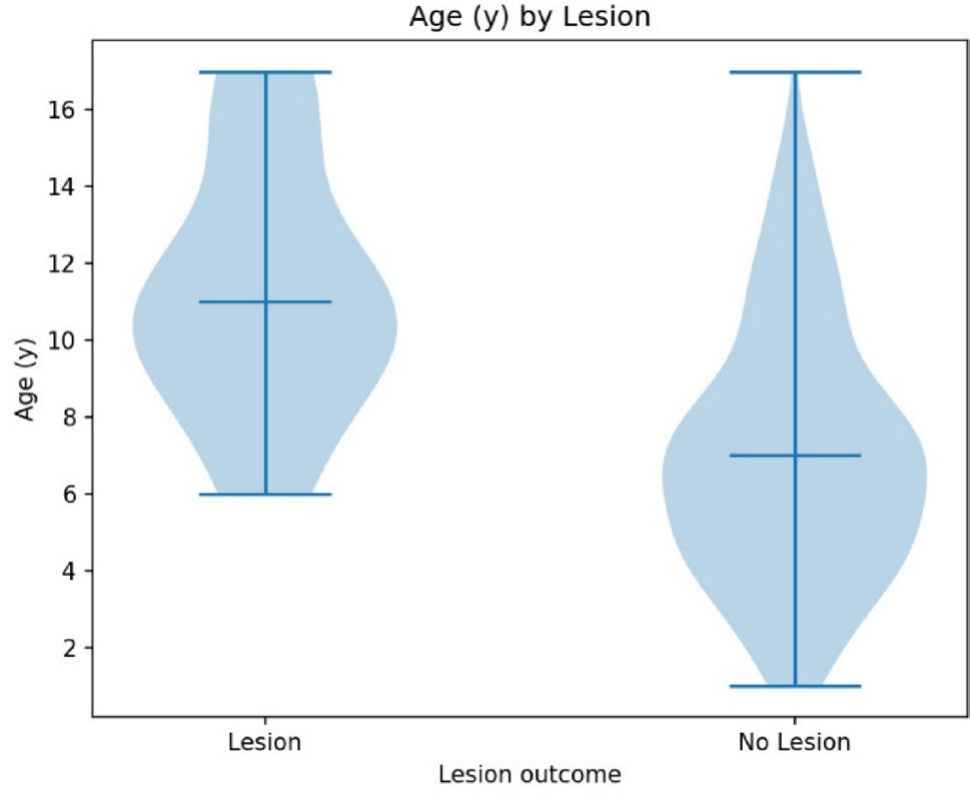
Violin plot of age distribution in groups the nerve lesion and without nerve lesion. Patients without lesion were significantly younger than patients with post-operative neurological deficit (*p* < 0.001)

Analysis of the association between patient age and the occurrence of postoperative neurological deficits demonstrated a significant age-related difference between groups. Children who developed a new postoperative neurological deficit were older at the time of surgery compared with neurologically unaffected patients. The Mann–Whitney U test confirmed this finding, revealing a highly significant difference between groups (*p* < 0.001). This pattern was visualized in a violin plot, which showed a distinct shift of the age distribution toward higher values in the deficit group (Fig. 6). Overall, the data indicate that increasing age is associated with a higher risk of postoperative neurological impairment following ESIN implantation.

#### Other complications and outcome

Besides neurological complications, a series of others was observed. Overall, we observed any complication in 53 patients (18.6%). Refractures occurred in 12 patients (4.2%), wound infection in 4 patients (1.4%). Soft tissue irritation was documented in 3 patients (1.1%) and 4 patients showed restricted range of motion after implant removal (1.4%). The other 281 patients showed free range of motion, all fractures showed radiological consolidation.

All neurological deficits were initially managed conservatively with clinical observation and follow-up. In cases of persistent symptoms, surgical revision with neurolysis was performed at the time of implant removal. Intraoperatively, this typically revealed either scar-related adhesions of the superficial radial nerve or mechanical irritation caused by the ESIN.

Other complications were treated according to standard clinical practice. Refractures were managed by re-osteosynthesis, wound infections were treated individually depending on severity, and restricted range of motion was addressed with physiotherapy.

Table 2 summarizes all complications observed in this cohort.

**Table 2:** Complications observed in our cohort

Complication	*n*	Total (%)
*Overall complications*	53	18.6
Refracture	12	4.2
Wound-infection	4	1.4
Restricted ROM	4	1.4
Soft tissue irritation	3	1.1
*Nerve injuries (overall*, *including trauma related)*	30	10.5
Radial nerve	21	7.4
Ulnar nerve	8	2.8
Median nerve	1	0.4
Transient postoperative nerve injury (SBRN)	9	3.2
Persistent postoperative nerve injury (SBRN)	6	2.1
*Nerve injury after ESIN implantation*		
Total	13	4,5
Radial nerve	12	4.2
Ulnar nerve	0	0
Median nerve	1	0.4
*Nerve injury after implant removal*		
Radial nerve	3	1.1
Ulnar nerve	0	0
Median nerve	0	0

Besides the neurological deficits,general complications were listed

## Literature research

 See Table 3.

**Table 3:** Summary of key studies reporting approach-related complications after elastic stable intramedullary nailing (ESIN) of pediatric forearm and radius fractures

Author	Title	Year	Journal	Study design	Complication
Lee et al. [22]	Incidence and Risk Factors for Extensor Pollicis Longus Rupture in Elastic Stable Intramedullary Nailing of Pediatric Forearm Shaft Fractures.	2016	J Pediatr Orthop	Retrospective cohort *n* = 17	Dorsal approach EPL rupture in 18%
Brooker et al. [20]	Rupture of the extensor pollicis longus tendon following dorsal entry flexible nailing of radial shaft fractures in children.	2014	J Child Orthop	Case series	Dorsal approach 9 EPL ruptures in a 5-year period Recommendation: Radial approach
Murphy et al. [21]	Extensor Tendon Injury Associated With Dorsal Entry Flexible Nailing of Radial Shaft Fractures in Children: A Report of 5 New Cases and Review of the Literature	2019	J Pediatr Orthop	Case series + review	Dorsal approach 5 EPL ruptures in a 10-year period
Varga et al. [8]	Intraoperative sonography may reduce the risk of extensor pollicis longus tendon injury during dorsal entry elastic intramedullary nailing of the radius in children.	2018	Medicine	Retrospective cohort *n* = 77	Dorsal approach, ultrasound-controlled No EPL rupture 0% with ultrasound Recommendation: Intraoperative sonography for monitoring entry point in dorsal approach
Cintean et al. [9]	Radial vs. Dorsal Approach for Elastic Stable Internal Nailing in Pediatric Radius Fractures – A 10 Year Review.	2022	J Clin Med	Retrospective comparative study *n* = 245	Dorsal approach (*n* = 201) 14 EPL ruptures (5.7%) Radial approach (*n* = 44) 4 SBRN-lesions (1.6%) Recommendation: Radial approach, especially in diaphyseal fractures
Nørgaard et al. [10]	Surgical approach for elastic stable intramedullary nail in pediatric radius shaft fracture: a systematic review.	2018	J Pediatr Orthop B	Systematic review	Dorsal approach EPL rupture 2.6% Radial approach SBRN-lesion (transient) 2.6%, (permanent) 0.3%
Makki et al. [13]	Elastic stable intramedullary nailing in paediatric forearm fractures: the rate of open reduction and complications.	2017	J Pediatr Orthop B	Retrospective review *n* = 102	Radial approach SBRN transient neuropathy 6.8% (all open reduction)
Kang et al. [14]	Elastic intramedullary nailing of paediatric fractures of the forearm.	2011	JBJS Br	Prospective cohort *n* = 90	Radial approach SBRN neuropathy in 2.2%
Winkelmann et al. [23]	Retrograde ESIN via posterior approach as cause of EPL rupture following pediatric forearm fractures	2025	Arch Orthop Trauma Surg	Retrospective cohort *n* = 352	EPL rupture Posterior approach major risk for EPL-ruptures (2.7%)
Kruppa et al. [6]	Low complication rate of elastic stable intramedullary nailing (ESIN) of pediatric forearm fractures: A retrospective study of 202 cases	2017	Medicine (Baltimore)	Retrospective study *n* = 202	EPL rupture 1.5% Radial approach for diaphyseal fractures, dorsal for more distal fractures. No information which approach caused rupture

## Discussion

In the present study, we analyzed complications and outcomes in 285 pediatric patients treated with elastic stable intramedullary nailing (ESIN) for fractures of the radius in a level I trauma center. A radial approach was used in all patients. Our data provide a differentiated analysis of neurological complications following radial entry ESIN of the radius, with particular emphasis on the superficial branch of the radial nerve (SBRN) as the anatomically relevant structure at risk.

Several studies have reported SBRN-related complications, for example, Fernandez et al. described 15 cases of SBRN lesions following ESIN for pediatric forearm fractures [12]. Our results demonstrate that new SBRN irritation after ESIN implantation occurred in approximately 5.3% of cases, with the majority being transient. Persistent hypesthesia in the SBRN distribution was observed in 2.1% of patients. These findings are in line with previously published data on lateral or radial entry techniques, where transient sensory disturbances of the SBRN have been reported in 2–5% of cases [9, 10, 13, 14]. In concordance with our data, Fernandez et al. reported lesions of the superficial radial branch in 2.7% of 553 patients, most often in the context of ESIN implantation or removal, and predominantly transient in nature [12].

Importantly, the rate of persistent deficits in our cohort was low (2.1%), supporting the notion that careful radial entry with direct visualization and protection of the nerve results mainly in temporary neurapraxia rather than permanent injury. Implant removal represented a second, independent risk for access-related nerve injury, with 1.1% persistent SBRN lesions, and should therefore be regarded as a separate surgical procedure with its own complication profile, requiring equal caution. Technical accurate implantation with precisely positioned and cut ends of the ESIN can help minimize intraoperative complications during the removal operation [15, 16].

Several factors influencing the risk of SBRN irritation were analyzed. Using the radial approach, the position of the entry point in relation to the distal epiphyseal plate appears to be a key determinant. Although statistical significance was narrowly missed (*p* = 0.055), a clear trend was identified showing that more proximally chosen entry points are associated with an increased risk of SBRN lesions.

Anatomically, the superficial branch of the radial nerve courses subcutaneously along the radial aspect of the distal forearm and becomes increasingly vulnerable with more proximal surgical approaches. This anatomical relationship is further complicated by considerable interindividual variability, as demonstrated in cadaveric studies, which have shown that no consistent safe zone can be defined for percutaneous approaches to the distal radius [17]. Importantly, these studies are almost exclusively based on adult specimens and therefore reflect fully developed radii without open growth plates.

In pediatric patients, however, the presence of an open distal radial physis fundamentally alters the surgical setting, as the entry point must be placed proximal to the growth plate to avoid physeal injury. Consequently, the surgical approach is inherently shifted into a region where the superficial radial nerve is already at increased risk, further limiting the applicability of cadaveric “safe zone” concepts derived from adult anatomy.

In addition, cadaveric investigations comparing different surgical techniques have demonstrated that even blunt dissection does not reliably reduce the risk of nerve injury, underscoring that anatomical variability rather than surgical technique alone is the predominant risk factor [18].

In this context, a proximally placed entry point may increase the likelihood of direct nerve irritation, traction injury, or postoperative scarring. Our data suggest a clinically relevant threshold at approximately 18 mm proximal to the distal radial epiphyseal plate, beyond which the risk of SBRN injury increases substantially. These findings support the recommendation to place the radial ESIN entry point as distal as safely possible while respecting the physis, as this appears to be one of the few modifiable factors influencing the risk of nerve injury.

The diameter of the nail in relation to the width of the intramedullary canal did not show a significant association with neurological complications. Similarly, ulnar involvement did not affect the occurrence of postoperative neurological complications, likely due to the anatomically distinct entry point for ulnar fixation at the olecranon.

Interestingly, our data showed a significantly increased risk of SBRN lesions in older patients (*p* < 0.001). This finding is in line with Martus et al., who reported a two-fold increase in overall complication rates in children older than 10 years, with neuropraxia occurring about five times more often in this age group [19]. This may be explained by more difficult reduction maneuvers in older children. In our cohort, the median age of patients requiring open reduction was 9 years, compared with 7 years in those treated by closed reduction. Makki et al. similarly reported transient SBRN neuropathy in 6.8% of cases, all of whom underwent open reduction [13]. In addition, detection of subtle sensory deficits may be more challenging in younger children, potentially leading to underreporting.

In contrast to dorsal entry techniques, which have repeatedly been associated with extensor pollicis longus (EPL) tendon rupture, no EPL injuries were observed in our exclusively radial-entry cohort [20, 21].

Cintean et al. reported an EPL rupture rate of 5.7% after dorsal approach, Lee et al. 18% [9, 22]. Although delayed EPL rupture after pediatric radius fractures is generally rare, Brooker et al. described an increased tendency following ESIN, either due to primary intraoperative injury or secondary attrition from protruding nail ends [20]. Winkelmann et al. found an EPL rupture rate of 2.7% in 352 patients and emphasized the dorsal approach as a major risk factor [23].

Rupture of the EPL results in loss of active thumb extension and therefore causes relevant impairment in activities of daily living [24, 25]. Most patients require secondary reconstruction using an extensor indicis proprius transfer. Even after successful reconstruction, measurable functional impairment has been reported using patient-reported outcome measures such as the QuickDASH [24, 25]. In pediatric cohorts, objective reductions in thumb strength and range of motion and decreased index finger extension have been observed, although subjective daily limitations were less pronounced [26]. In contrast, SBRN injury is usually limited to sensory disturbance. While chronic neuropathic pain may rarely develop, most cases present as transient paresthesia of the radial half of the dorsal hand and rarely interfere with daily activities [27].

These findings support the growing body of evidence suggesting that dorsal entry is associated with a risk of relevant tendon complications, whereas radial entry predominantly carries a risk of mostly transient sensory nerve irritation. Table 3 provides an overview of key studies reporting approach-related complications after ESIN in pediatric forearm fractures. The summarized data consistently demonstrate a higher rate of extensor tendon injuries associated with dorsal entry techniques, whereas radial approaches are predominantly linked to transient sensory disturbances of the superficial radial nerve.

### Limitations

The limited number of neurological events restricts definitive conclusions. Another limitation is the retrospective design, with reliance on chart documentation for neurological findings and functional outcomes. Prospective studies with standardized neurological assessment and defined radiographic entry-point measurements are needed to further define an optimal safe zone for radial ESIN entry.

## Conclusion

Radial entry ESIN for pediatric radius fractures is associated with a low rate of predominantly transient neurological complications. Entry point position and patient age appear to influence the risk of superficial radial nerve injury. In contrast to dorsal entry techniques, no extensor pollicis longus tendon ruptures were observed. The radial approach therefore represents a clinically favorable option.

## Data Availability

The data underlying this study are not publicly available due to patient privacy and institutional regulations but are available from the corresponding author on reasonable request.
